# Similar-Case-Based Optimization of Beam Arrangements in Stereotactic Body Radiotherapy for Assisting Treatment Planners

**DOI:** 10.1155/2013/309534

**Published:** 2013-11-02

**Authors:** Taiki Magome, Hidetaka Arimura, Yoshiyuki Shioyama, Katsumasa Nakamura, Hiroshi Honda, Hideki Hirata

**Affiliations:** ^1^Department of Health Sciences, Graduate School of Medical Sciences, Kyushu University, Fukuoka 8128582, Japan; ^2^Department of Radiology, The University of Tokyo Hospital, Tokyo 1138655, Japan; ^3^Department of Health Sciences, Faculty of Medical Sciences, Kyushu University, Fukuoka 8128582, Japan; ^4^Division of Quantum Radiation Science, Department of Health Sciences, Faculty of Medical Sciences, Kyushu University, 3-1-1, Maidashi, Higashi-ku, Fukuoka 812-8582, Japan; ^5^Department of Heavy Particle Therapy and Radiation Oncology, Graduate School of Medical Sciences, Kyushu University, Fukuoka 8128582, Japan; ^6^Department of Clinical Radiology, Graduate School of Medical Sciences, Kyushu University, Fukuoka 8128582, Japan

## Abstract

*Objective*. To develop a similar-case-based optimization method for beam arrangements in lung stereotactic body radiotherapy (SBRT) to assist treatment planners. *Methods*. First, cases that are similar to an objective case were automatically selected based on geometrical features related to a planning target volume (PTV) location, PTV shape, lung size, and spinal cord position. Second, initial beam arrangements were determined by registration of similar cases with the objective case using a linear registration technique. Finally, beam directions of the objective case were locally optimized based on the cost function, which takes into account the radiation absorption in normal tissues and organs at risk. The proposed method was evaluated with 10 test cases and a treatment planning database including 81 cases, by using 11 planning evaluation indices such as tumor control probability and normal tissue complication probability (NTCP). *Results*. The procedure for the local optimization of beam arrangements improved the quality of treatment plans with significant differences (*P* < 0.05) in the homogeneity index and conformity index for the PTV, V10, V20, mean dose, and NTCP for the lung. *Conclusion*. The proposed method could be usable as a computer-aided treatment planning tool for the determination of beam arrangements in SBRT.

## 1. Introduction

Stereotactic body radiotherapy (SBRT) is a sophisticated technique to improve survival rates for early-stage lung cancers [1–4]. SBRT can be used to deliver highly conformal doses to tumors while minimizing radiation doses to surrounding organs at risk (OAR) and normal tissues with steep dose gradients. Radiotherapy treatment planning (RTP) is one of the most important procedures for SBRT, which is determined by treatment planners in time-consuming iterative manners. In particular, it is essential to determine an appropriate beam arrangement, which generally consists of a large number of coplanar and non-coplanar beams [5, 6]. The beam arrangement includes not only beam directions but also nominal beam energies, collimator angles, and beam weights. Many researchers have investigated automated methods for beam angle optimization (BAO). Li and Lei [7] developed a DNA-based genetic algorithm to solve the BAO problem in coplanar directions for intensity-modulated radiation therapy (IMRT) planning. De Pooter et al. [8] investigated an optimization method for non-coplanar beams based on the Cycle algorithm for SBRT of liver tumors. Meyer et al. [9] developed an automated method for the selection of non-coplanar beams by use of the cost function based on radiation absorption in normal tissue and OAR for three-dimensional conformal radiotherapy. Although the above-cited authors stated that the treatment planning time could be reduced by using their BAO algorithms, there is still room for improvement in the routine clinical use of BAO methods.

The usefulness of similar cases in the field of radiation oncology has been shown in several studies. Commowick and Malandain [10] used a similar image in a database for the segmentation of critical structures. Chanyavanich et al. [11] developed new prostate IMRT plans based on a similar case. Mishra et al. [12] investigated the case-based reasoning approach to determine the most appropriate dose plan for prostate cancer patients. Schlaefer and Dieterich [13] showed the feasibility of case-based beam generation for robotic radiosurgery. These studies motivated us to adopt some novel strategies to determine the clinically usable beam arrangements for SBRT based on past similar cases.

In our earlier research, we investigated the usefulness of similar cases in an RTP database for the determination of beam arrangements in SBRT [14, 15]. However, our previous work had a problem that the beam direction of the past similar case may not be optimal for a new case. Therefore, there is a potential to improve the accuracy and efficiency of the determination of beam arrangements based on the combination of similar-case-based beam arrangement and the BAO algorithm. Our purpose in the present study was to develop a similar-case-based optimization method for beam arrangement in lung SBRT for assisting treatment planners. We evaluated our method by comparing the method without and with an optimization step of beam arrangements and also by comparing the most usable beam arrangements based on the proposed method with original beam arrangements based on a manual method.

## 2. Materials and Methods

**Figure 1:**
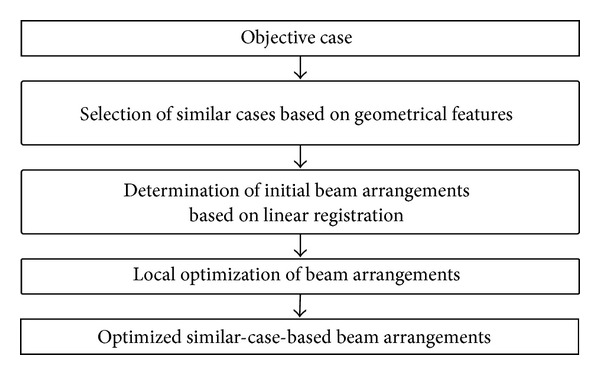
Overall scheme of our similar-case-based optimization method for beam arrangements in SBRT.

Figure 1 presents the overall scheme of the proposed method, which consists mainly of three steps. First, cases that are similar to an objective case were automatically selected based on geometrical features related to structures such as the location, size, and shape of the planning target volume (PTV), lung, and spinal cord. Second, the initial beam arrangements of the objective case were determined by registering cases that are similar to the objective case in terms of lung regions, using a linear registration technique, that is, an affine transformation [16]. Finally, the beam directions of the objective case were locally optimized based on the cost function, which takes into account the radiation absorption in normal tissues and OAR.

### 2.1. Clinical Cases

The institutional review board of our university hospital approved this retrospective study. We selected 96 patients (ages: 42–92 years; median: 76 years) with lung cancer (right lung: 52 cases, left lung: 44 cases) who were treated with SBRT from November 2003 to April 2010. Sixty patients were males and 36 patients were females. Their mean effective PTV dia. was 4.0 ± 0.7 cm. All patients were fixed with a body cast system composed of a thermoplastic body cast, a vacuum pillow, arm and leg supports, and a carbon plate [17]. Experienced radiation oncologists performed the treatment planning on an RTP system by using a pencil beam convolution algorithm (Eclipse versions 6.5 and 8.1; Varian Medical Systems Inc., Palo Alto, CA, USA). The contours of the gross tumor volumes of lung cancers were manually outlined on planning computed tomography (CT) images acquired from a four-channel detector CT scanner (Mx 8000; Philips, Amsterdam, The Netherlands). Each CT slice had a matrix size of 512 × 512, a slice thickness of 2.0–5.0 mm, a pixel size of 0.78–0.98 mm, and a stored bit of 12. The internal target volume (ITV) was determined individually according to the internal respiratory motion, which was measured with an X-ray simulator (Ximatron; Varian Medical Systems). The setup margins surrounding the ITV were 5 mm in all directions. Seven to eight static beams—including beams in the coplanar and non-coplanar directions—were arranged depending on each patient. All patients received a dose of 48 Gy in four fractions at the isocenter with 4, 6, or 10 MV beams on linear accelerators (Clinac 21EX; Varian Medical Systems).

 All cases were randomly separated into three datasets: a dataset of the RTP database including 81 cases (right lung: 46 cases, left lung: 35 cases), a dataset of five training cases (right lung: 3 cases, left lung: 2 cases), and a dataset of 10 test cases (right lung: 3 cases, left lung: 7 cases). The five training cases were used to determine the parameters for the selection of similar cases, and the 10 test cases were used for the evaluation of our method.

### 2.2. Selection of Similar Cases Based on Geometrical Features

Five cases that were similar to an objective case were automatically selected based on geometrical features from the treatment planning point of view. The geometrical features were the PTV location, the PTV shape, the PTV size, the lung volume, and the geometrical relationship between the PTV and spinal cord in making an SBRT treatment plan for lung cancer. The five most similar cases were defined as the cases who had first to fifth shortest distances to the objective case in a feature space. The RTP database was searched for the five cases most similar to the objective case by considering the weighted Euclidean distance of geometrical feature vectors between the objective case and each case in the RTP database. The weighted Euclidian distance *d*_image_ was calculated by the following equation:

(1) $$\begin{array}{c} d_{\text{image}}=\sqrt{\overset{G}{\underset{i=1}{\sum }}w_{i}{\left(A_{i}-B_{i}\right)}^{2}}, \end{array}$$

where *G* is the number of geometrical features, *w*_*i*_ is the weight of the *i*th geometrical feature, *A*_*i*_ is the *i*th geometrical feature for the objective case, and *B*_*i*_ is the *i*th geometrical feature for each case in the RTP database. Note that each geometrical feature was divided by the standard deviation of all 81 cases in the RTP database for normalizing the range of each feature value.  Table 1 shows 10 geometrical features which were used for the selection of similar cases.

The geometrical features were calculated by the following definitions. The PTV centroid was calculated in a fixed reference coordinate system. Each case in the RTP database was registered to a reference case based on the linear registration technique, that is, affine transformation [16]. Feature points for the registration were automatically set to the vertices of a circumscribed parallelepiped of a lung including left and right lung regions as follows. First, the minimum and maximum *x*, *y*, and *z* coordinates, *x*_min⁡_, *x*_max⁡_, *y*_min⁡_, *y*_max⁡_, *z*_min⁡_, and *z*_max⁡_, were obtained in the original coordinate system of the planning CT image from the lung segmented by a treatment planner, and then the six planes *x* = *x*_min⁡_, *x* = *x*_max⁡_, *y* = *y*_min⁡_, *y* = *y*_max⁡_, *z* = *z*_min⁡_ and *z* = *z*_max⁡_ were determined as those of the circumscribed parallelepiped. Finally, two vertices of the circumscribed parallelepiped of the lung region (*x*_min⁡_, *y*_min⁡_, *z*_min⁡_) and (*x*_max⁡_, *y*_max⁡_, *z*_max⁡_) were used as feature points for the determination of parameters in the affine transformation matrix. In this study, we used a special case of an affine transformation including only the translation and scaling based on two feature points. The reason for this was to reduce the calculation time for finding the feature points of the lung regions. The effective diameter was defined as the diameter of a sphere with the same volume as the PTV. The sphericity was defined as a roundness of the PTV and was given by a ratio of the number of logical AND voxels between the PTV and its equivalent sphere with the same centroid and volume as the PTV to the number of PTV voxels. Lung dimensions were defined as three side-lengths of the circumscribed parallelepiped of the lung regions in the left-right (LR), anterior-posterior (AP), and superior-inferior (SI) directions. The distance between the PTV and spinal cord was measured between the centroid of the PTV and that of the spinal cord in the isocenter axial plane. The angle from the spinal cord to the PTV was defined in the two-dimensional coordinate system with the origin at the centroid of the spinal cord in the isocenter axial plane and ranged from −*π* (clockwise) to *π* (counterclockwise) for a baseline of the posterior-anterior direction.

 The weights of the geometrical features were needed in order to determine the features' importance. Therefore, each institute should determine the appropriate weights of the geometrical features based on their own philosophy or policy of treatment planning when applying the proposed method to their own databases. In the present study, the weights of the geometrical features were empirically set as follows: based on our institution's treatment planning policy by using the five training cases with a trial-and-error procedure so that cases more similar to the objective case could be selected in terms of appearance relevant to the features. As a result, the geometrical feature weights were the PTV centroid = 0.3, effective dia. of the PTV = 0.1, sphericity of the PTV = 0.1, lung dimension = 0.3, distance between the PTV and spinal cord = 1.0, and angle from spinal cord to the PTV = 1.0. The weights for geometrical features were normalized as the sum of the weights was 1.0 when the similarity measure was calculated in our system.

### 2.3. Determination of Initial Beam Arrangements Based on the Linear Registration

**Figure 2:**
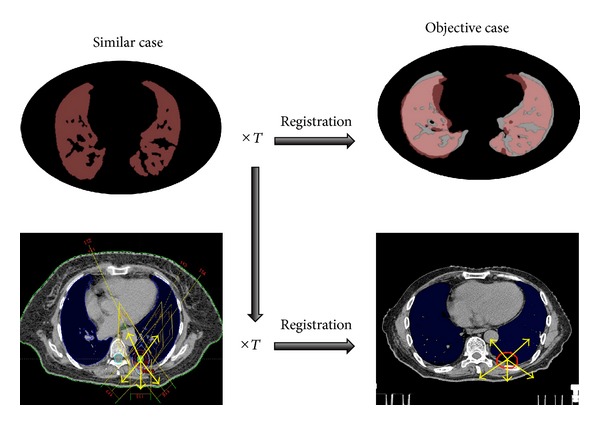
The determination of beam arrangements based on a linear registration technique. *T* is a transformation matrix.

Figure 2 illustrates the determination of initial beam arrangements based on the linear registration technique. Five beam arrangements, each of which had seven or eight beam directions, for an objective case were automatically determined by the registration of five similar cases with the objective case in terms of lung regions using a linear registration technique, that is, affine transformation [16]. Please note that the beam directions are determined indirectly by the registration of the lung regions, because the linear registration maps straight lines, that is, beam angles, to straight lines. First, we calculated the affine transformation matrix *T* to register the lung regions of each similar case with those of the objective case based on feature points, which were automatically selected for the registration in vertices of the circumscribed parallelepiped of the lung regions. Second, a beam direction, that is, beam direction vector, based on a gantry angle *θ* and couch angle *ϕ* was transformed from a spherical polar coordinate system to a Cartesian coordinate system as the unit direction vector (*a*, *b*, *c*) as follows:

(2) $$\begin{array}{c} \left(\begin{array}{c} a\\ b\\ c \end{array}\right)=\left(\begin{array}{c} \sin\theta \cos\phi \\ -\cos\theta \\ \sin\theta \sin\phi \end{array}\right). \end{array}$$

Third, each beam direction vector of the similar case in the Cartesian coordinate system was modified by using the same affine transformation matrix *T* as a registration in terms of lung regions. Finally, the resulting direction vector (*a*′, *b*′, *c*′) in the Cartesian coordinate system was converted into the spherical polar coordinate system as gantry angle *θ*′ and couch angle *ϕ*′ as follows:

(3) $$\begin{array}{c} \theta^{\prime }=\text{ta}{\text{n}}^{-1}\left(\frac{\sqrt{a^{\prime 2}+c^{\prime 2}}}{-b^{\prime }}\right),\\ \phi^{\prime }=\text{ta}{\text{n}}^{-1}\left(\frac{c^{\prime }}{a^{\prime }}\right). \end{array}$$

### 2.4. Local Optimization of Beam Arrangements

The beam directions of the objective case were locally optimized based on the cost function, which takes into account the radiation absorption in normal tissues and OAR [9]. Although Meyer et al. [9] developed the cost function for a global optimization of beam arrangements, we used the Meyer cost function for the local optimization of each beam direction. The cost function *C*_*θ*,*ϕ*_ of the beam with gantry angle *θ* and couch angle *ϕ* was defined as follows:

(4) $$\begin{array}{c} C_{\theta ,\phi }=C_{\theta ,\phi }\left(\text{PTV}\right)+\sum_{k}w_{k}C_{\theta ,\phi }\left(\text{OA}{\text{R}}_{k}\right), \end{array}$$

where *C*_*θ*,*ϕ*_(PTV) represents a dose absorption in normal tissue until X-ray beams reach the PTV surface, *C*_*θ*,*ϕ*_(OAR_*k*_) is a term for the irradiation of *k*th OAR, and *w*_*k*_ is a weight for the *k*th OAR. The first term *C*_*θ*,*ϕ*_(PTV) is determined by the following equation:

(5) $$\begin{array}{c} C_{\theta ,\phi }\left(\text{PTV}\right)=1-\exp\left(-\mu d_{\theta ,\phi }\left(\text{PTV}\right)\right), \end{array}$$

where *μ* is a linear attenuation coefficient in water, and *d*_*θ*,*ϕ*_(PTV) is the mean depth in cm from the body surface to the PTV surface. In the present study, the *μ* values for the 4, 6, and 10 MV beams were set to 0.05730, 0.05271, and 0.03859 cm^−1^, respectively. The second term for the *k*th OAR *C*_*θ*,*ϕ*_(OAR_*k*_) is defined as follows:

(6) $$\begin{array}{c} C_{\theta ,\phi }\left(\text{OA}{\text{R}}_{k}\right)=\lambda v_{\theta ,\phi }\left(\text{OA}{\text{R}}_{k}\right)+\left(1-\lambda \right)\exp\left(-\mu d_{\theta ,\phi }\left(\text{OA}{\text{R}}_{k}\right)\right), \end{array}$$

where *v*_*θ*,*ϕ*_(OAR_*k*_) is an irradiated fractional volume of the *k*th OAR, *d*_*θ*,*ϕ*_(OAR_*k*_) is the mean depth from the body surface to the *k*th OAR surface, and *λ* is a parameter to control the relative significance of the first and second terms. The term of exp(−*μd*_*θ*,*ϕ*_(OAR_*k*_)) represents the number of incident photons in the *k*th OAR. The beam directions were locally optimized in the ascending order of the cost functions of the initial angles.

**Figure 3:**
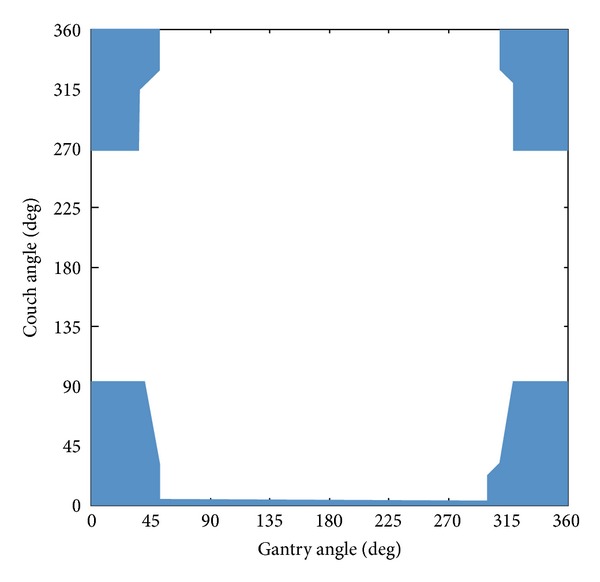
The available beam direction space, which was used in past cases including the RTP database. The blue area represents the applicable combination of the gantry and couch angles.

 Each beam direction was locally optimized in the range of ±*α* degree with the interval of *β* degree. Here, the beam directions were constrained by following three conditions: (i) although the non-coplanar beams can be arranged with the change of gantry and couch angles, the coplanar beams can be shifted only in the direction of gantry rotation; (ii) the interval with the optimized beam directions was greater than or equal to *γ* degree, and (iii) the available beam direction space was limited to the space which was used in past cases including the RTP database (as shown in Figure 3) to avoid the collision of the patient with a gantry head. In this study, the lung and spinal cord were incorporated as OAR in the cost function, and both weights (for lung and spinal cord) were empirically set to 5 by using the training case dataset. The parameters for the local optimization of beam arrangement *λ*, *α*, *β*, and *γ* were empirically set to 0.6, 4°, 2°, and 40°, respectively, by using the training case dataset. Although the parameters for the local optimization of beam arrangements were empirically set based on the preferences of our institution, each institute can determine appropriate parameters based on their own philosophy or policy of treatment planning in the same way as the geometrical feature weights. Each optimal beam direction was defined as the direction of the beam which has the lowest cost value in the beam directions of the local range.

### 2.5. Evaluation of Beam Arrangements Using Planning Evaluation Indices

The five patterns of beam arrangements determined by the proposed method were evaluated by manually making five plans based on the beam arrangements with other planning parameters (nominal beam energies, collimator angles, beam weight, etc.) derived from treatment plans of similar cases in a radiation treatment planning system.  Table 2 shows 11 planning evaluation indices for the evaluation of our method. The most usable plan of the objective case was selected by sorting the five plans based on an RTP evaluation measure with these 11 planning evaluation indices, which was the Euclidean distance in a feature space between each plan and an ideal plan.

 In this study, the ideal plan was assumed to produce a uniform irradiation with a prescribed dose in the PTV and no irradiation in the surrounding OAR and normal tissues. The usefulness of each plan was estimated by the following Euclidean distance *d*_plan_ of the planning evaluation vector between the ideal plan and each plan determined by a similar case, which was considered as the RTP evaluation measure:

(7) $$\begin{array}{c} d_{\text{plan}}=\sqrt{\overset{J}{\underset{j=1}{\sum }}{\left(X_{j}-Y_{j}\right)}^{2}}, \end{array}$$

where *J* is the number of planning evaluation indices, *X*_*j*_ is the *j*th planning evaluation index for the ideal plan, and *Y*_*j*_ is the *j*th planning evaluation index for the plan based on the five most similar cases. Each planning evaluation index was normalized by the standard deviation in the same manner as the geometrical features based on the RTP database of 81 cases with lung cancer.

 The planning evaluation indices for the ideal plan were defined as follows: D95 = 48 Gy (prescribed dose), homogeneity index (HI) = 1.0, conformity index (CI) = 1.0, tumor control probability (TCP) = 100%, V5 = 0%, V10 = 0%, V20 = 0%, mean lung dose = 0 Gy, normal tissue complication probability (NTCP) for the lung = 0%, spinal cord maximum dose = 0 Gy, and the NTCP for the spinal cord = 0%. We evaluated similar-case-based beam arrangements suggested by the proposed method using (7) based on the Euclidean distance of the 11 planning evaluation indices. Although we can apply weights to planning evaluation indices based on planners' preferences, we decided to give a constant weight to each planning evaluation index in this study.

 The planning evaluation indices for the PTV calculated in this study were the D95, HI, CI, and TCP. The D95 was defined as the minimum dose in the PTV that encompasses at least 95% of the PTV. The HI was calculated as the ratio of the maximum dose to the minimum dose in the PTV [18]. The CI was the ratio of the treated volume to the PTV. The treated volume was defined as the tissue volume that is intended to receive at least the selected dose and is specified by the radiation oncologist as being appropriate to achieve the purpose of the treatment [19]. In the present study, the treated volume was defined as the volume receiving the minimum PTV dose. The TCP was estimated based on a linear-quadratic (LQ) model according to a Poisson distribution by consideration of the radiosensitivity variation and nonuniform dose distribution [20, 21]. We used parameters for TCP calculation which were obtained from Kanai et al. [22] in patients with lung cancers. The details of the calculation of the TCP were described previously [15].

 The planning evaluation indices for normal tissues, that is, the lung and spinal cord, were calculated as described below. For the lung volume, which was defined as total lung volume minus PTV, a V5, V10, V20, and mean dose were calculated. The V*k* was defined as a percentage of the total lung minus PTV receiving ≥*k* Gy. The maximum dose for the spinal cord was also calculated. Moreover, the NTCP values for the lung and spinal cord were calculated using the Lyman-Kutcher-Burman model [23, 24]. The fitting parameter values for NTCP calculation were obtained from Burman et al. [25] More details for the calculation of the NTCP can be found in our previous work [15].

### 2.6. Assessment of the Proposed Method

The proposed method was assessed with an RTP database including 81 cases with lung cancer (right lung: 46 cases, left lung: 35 cases) and 10 test cases (right lung: 3 cases, left lung: 7 cases), which were randomly chosen from all 96 cases. The 10 test cases were not included in the RTP database of 81 cases and were not used as five training cases for the determination of the weights of geometrical features. The similar cases were selected from cases that have ipsilateral lung cancers with the test case. The effectiveness of the combination method for the determination of the initial beam arrangement based on similar cases and the local optimization of the beam arrangement was evaluated by comparing the planning evaluation indices of 50 plans (5 plans × 10 test cases) with and without the local optimization of the beam arrangement. In addition, the most usable beam arrangements determined based on the RTP evaluation measure were retrospectively compared with the original beam arrangements, which were used in clinical practice in 10 test cases. The same beam weights and wedges of the similar case were used for the plan with the beam arrangement determined by our method. The irradiation fields were adjusted to the tumor using a multileaf collimator (millennium 120 MLC; Varian Medical Systems) with an additional margin of 5 mm around the PTV.

## 3. Results

**Figure 4:**
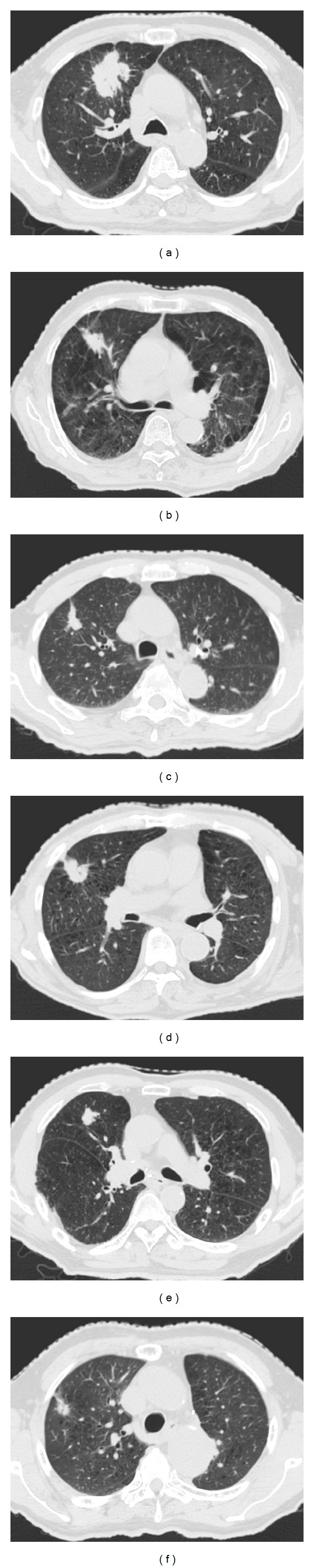
The first to fifth most similar cases to the objective case obtained by the proposed method: (a) an objective case and ((b)–(f)) the first to fifth most similar cases.

Figure 4 shows an objective case (a) and the first to fifth most similar cases ((b)–(f)) to the objective case. The similar cases geometrically resemble the objective case (Figure 4(a)), especially in terms of the geometrical relationship between the tumor and the spinal cord, because we gave greater weights to the geometrical features related to the spinal cord.

**Figure 5:**
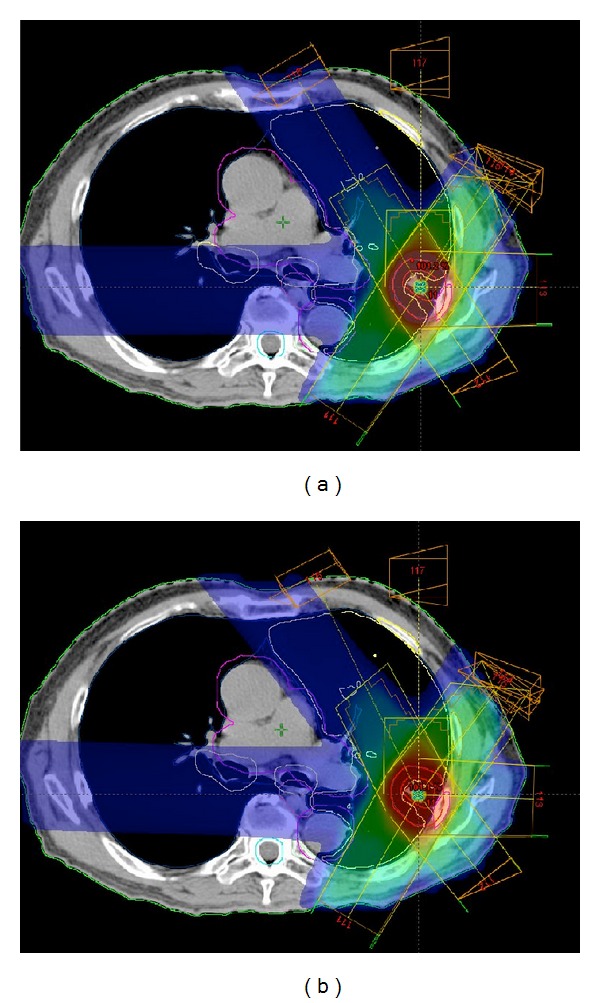
Dose distributions determined by beam arrangements without and with the beam angle optimization step: (a) the similar-case-based beam arrangements based on the linear registration technique, and (b) the optimized similar-case-based beam arrangement determined by the local optimization of each beam direction.

Figure 5 illustrates the similar-case-based beam arrangements based on a linear registration technique (a) and the optimized similar-case-based beam arrangement after the local optimization of each beam direction (b). These plans consisted of seven beams with gantry angle and couch angle of (214, 0), (146, 0), (90, 0), (40, 333), (35, 32), (325, 328), and (328, 90) in Figure 5(a) and (210, 0), (142, 0), (92, 0), (40, 333), (39, 28), (329, 324), and (328, 90) in Figure 5(b). Although the lateral beam passed the spinal cord in the beam arrangement before the local optimization step in Figure 5(a) (spinal cord max. dose: 3.13 Gy), the optimized beam arrangement avoids the spinal cord in Figure 5(b) (spinal cord max. dose: 1.68 Gy).  Table 3 shows mean ± standard deviation (SD) of the planning evaluation indices in 50 treatment plans of 10 test cases obtained from the dose distributions of similar-case-based beam arrangements without and with the beam direction optimization. The procedure for the local optimization of beam arrangements improved the quality of the treatment plans with significant differences (*P* < 0.05) in both the homogeneity index and conformity index for the PTV and V10, V20, mean dose, and NTCP for the lung.

**Figure 6:**
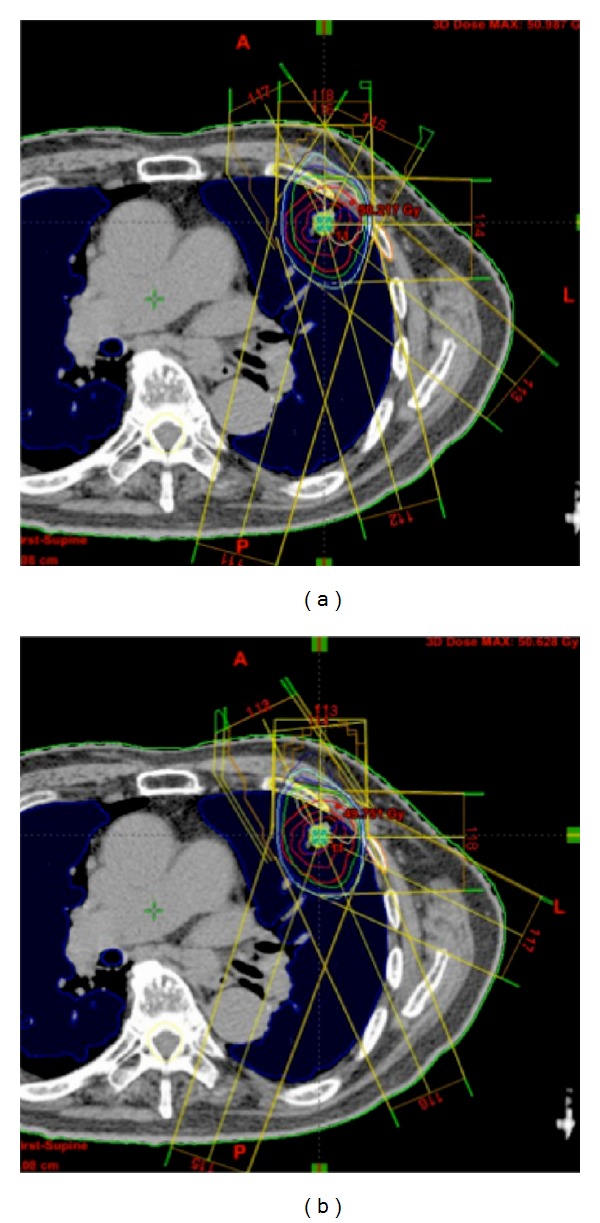
(a) A plan obtained from the original beam arrangement, which was determined by an experienced radiation oncologist. (b) A plan obtained by the most usable beam arrangement based on the proposed method.

**Figure 7:**
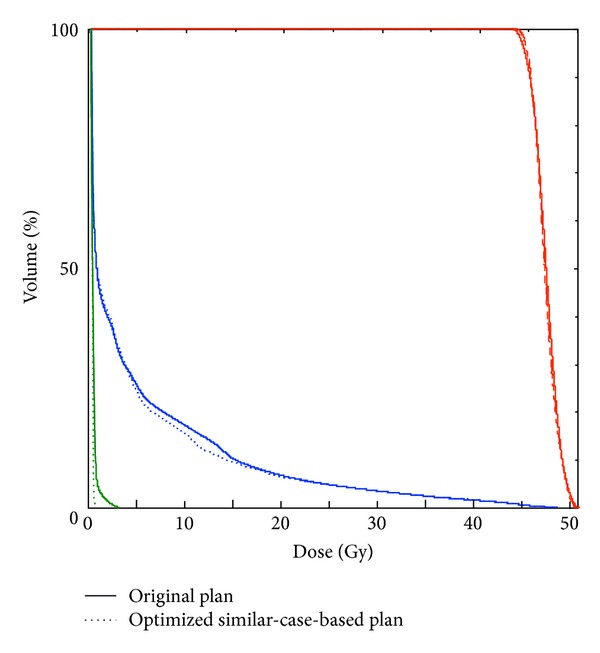
Dose-volume histograms obtained from the original beam arrangement in Figure 6(a) (solid lines) and optimized similar-case-based beam arrangement in Figure 6(b) (dotted lines). Planning target volume (red), lung (blue), and spinal cord (green).

Figure 6 shows dose distributions of the original plan (Figure 6(a)) and the most usable plan (Figure 6(b)) determined by the RTP evaluation measure.  Figure 7 provides dose-volume histograms (DVHs) for the case shown in Figure 6. In terms of the PTV, the similar-case-based plan has a DVH curve that is almost the same as that of the original plan. However, the similar-case-based plan resulted in better sparing of spinal cord and lung regions compared to the original plan.  Table 4 shows mean ± SD of the planning evaluation indices in 10 test cases obtained from the dose distributions produced by original beam arrangements and similar-case-based beam arrangements of the most usable plans determined by the RTP evaluation measure. The proposed method may provide usable beam directions that are not significantly different from those obtained with the original beam arrangements (*P* > 0.05) in terms of the 10 planning evaluation indices out of 11 indices. The mean value of D95 was significantly improved based on the proposed method compared to that of the original beam arrangements (*P* = 0.029).

## 4. Discussion

We have shown the feasibility of our similar-case-based optimization method for the determination of beam arrangements in SBRT. In general, the appropriate beam arrangement in lung SBRT has varied with an institution's situation. In terms of the number of beams, Takayama et al. [5] reported that they routinely used 5 to 10 beams with coplanar and non-coplanar directions for lung SBRT in order to make a homogenous target dose distribution, while avoiding high doses to normal tissues. Liu et al. [26] found that the optimal number of beams for lung SBRT was 13 to 15 with coplanar and non-coplanar directions. A large number of beams increase the required treatment time, which should be as short as possible to reduce the patient's burden. Moreover, an available beam direction space is restricted by the size of the gantry and an immobilizer. In our method, the beam arrangement was automatically determined based on the past similar case, followed by the local optimization of each beam direction. Therefore, the proposed method could adjust an institution's specific situation by replacing the RTP database.

One of the most difficult problems in RTP is the patient-specific tradeoff between the benefit to a tumor and the risk to surrounding normal tissues. Therefore, treatment planners should select a plan that is most suitable for each individual patient, from a set of optimal plans. In our method, treatment planners can select a plan considered the best for the patient from among several plans based on similar cases in the RTP database with the knowledge of experienced treatment planners. Although the RTP is a time-consuming task—especially for the less experienced treatment planners—the combination of similar cases and the BAO algorithm may reduce both the workload for treatment planners and the interplanner variability of treatment plans.

As a result of the local optimization of beam arrangements, the planning evaluation indices were improved with significant differences as shown in Table 3. Although the improvement of the planning evaluation indices may not have a great impact on a clinical outcome from dosimetric point of view (e.g., mean lung V20 reduced from 4.34% to 4.25%), the step of the local optimization of beam arrangements gave robustness to the proposed method. As shown in Figure 5, the optimized beam arrangement avoided OARs, even if the beam arrangement of the past similar case may not be suitable for a new case. Therefore, the treatment planning time for the manual corrections of beam arrangements could be reduced based on the proposed method with the BAO. 

 It would be difficult to compare the planning evaluation indices between our results and other BAO literatures [7–9] due to a difference of clinical cases. Although Liu et al. applied the BAO algorithm to IMRT treatment plans for stage III lung cancers [27], to the best of our knowledge, there are no literatures which applied the BAO algorithm to the SBRT treatment plans for early stage lung cancers with 48 Gy in four fractions. In addition, the commercialized BAO software (Eclipse version 8.1; Varian Medical Systems) was not used for the comparison with the proposed method, because the beam directions obtained by the BAO software might be located at the outer side of the available beam direction space as shown in Figure 3.

 It is important to investigate how long it takes for treatment planners, who are not experienced, to make SBRT plans with and without the proposed method. In addition, it is necessary to evaluate which type of case the proposed method is more effective with respect to the planning time, simple cases (frequent cases, whose tumors are located at far from the OARs), and the complex cases (rare cases, whose tumors are located at close to the OARs). Therefore, we should compare the treatment planning time with and without the proposed method and also between simple and complex cases in future work.

 There are some limitations of this method. First of all, the proposed method has a possibility to be trapped into local minima of the cost function in the local optimization step of beam arrangement, because each beam direction is locally optimized with the initial angle, which is derived from the similar case. In other words, our method depends on the quality of treatment plans in the RTP database. Although the RTP database used here consisted of treatment plans developed by senior experienced radiation oncologists at our institution, we should collect many more treatment plans with clinical outcomes to improve the quality of the RTP database in future work. In addition, there are several parameters to adjust in our method. In the present study, we empirically determined the parameters based on the institution's policy of treatment planning by using the five training cases with a trial-and-error procedure. In the future, we will need to optimize the parameters by using a larger number of cases.

## 5. Conclusion

We developed a similar-case-based optimization method for beam arrangements in lung SBRT for assisting treatment planners. The local BAO algorithm improved the quality of treatment plans with significant differences (*P* < 0.05) in the homogeneity index and conformity index for the PTV, V10, V20, mean dose, and NTCP for the lung. Moreover, the proposed method may provide usable beam arrangements which are not significantly different from the original beam arrangements (*P* > 0.05) in terms of the 10 planning evaluation indices. The mean value of D95 was significantly improved based on the proposed method compared to that of the original beam arrangements (*P* = 0.029). Therefore, our system will be useful for treatment planners, and thus, the quality and efficiency of radiotherapy would be improved.

## Figures and Tables

**Table 1:** Geometrical features for selection of similar cases.

PTV location	PTV centroid in LR direction
	PTV centroid in AP direction
	PTV centroid in SI direction

PTV shape	Effective diameter of PTV
	Sphericity of PTV

Lung dimension	Lung length in LR direction
	Lung length in AP direction
	Lung length in SI direction

Spinal cord position	Distance between PTV and spinal cord in isocenter plane
	Angle from spinal cord to PTV in isocenter plane

PTV: planning target volume; LR: left-right; AP: anterior-posterior; SI: superior-inferior.

**Table 2:** Planning evaluation indices used for searching usable plans.

PTV	D95 (Gy)
	Homogeneity index
	Conformity index
	TCP (%)

Lung*	V5 (%)
	V10 (%)
	V20 (%)
	Mean lung dose (Gy)
	NTCP_lung_ (%)

Spinal cord	Maximum dose (Gy)
	NTCP_spinal cord_ (%)

*Planning evaluation indices for lung were calculated in a lung volume with subtraction of a planning target volume (PTV). TCP: tumor control probability; NTCP: normal tissue complication probability.

**Table 3:** Mean ± standard deviation of the planning evaluation indices in 50 treatment plans of 10 test cases obtained from the dose distributions of similar-case-based beam arrangements without and with the beam direction optimization.

	Similar-case-based beam arrangement	Optimized similar-case-based beam arrangement	*P* value
PTV			
D95 (Gy)	45.4 ± 1.04	45.5 ± 0.82	0.135
Homogeneity index	1.15 ± 0.05	1.14 ± 0.05	0.023
Conformity index	1.83 ± 0.27	1.81 ± 0.27	0.001
TCP (%)	95.9 ± 0.48	96.0 ± 0.33	0.259
Lung			
V5 (%)	15.7 ± 5.62	15.6 ± 5.68	0.248
V10 (%)	10.0 ± 4.05	9.83 ± 4.03	< 0.001
V20 (%)	4.34 ± 1.61	4.25 ± 1.57	< 0.001
Mean dose (Gy)	3.08 ± 1.01	3.05 ± 1.00	< 0.001
NTCP_lung_ (%)	9.25 × 10^−3^ ± 1.86 × 10^−2^	8.29 × 10^−3^ ± 1.63 × 10^−2^	0.020
Spinal cord			
Maximum dose (Gy)	7.44 ± 5.90	7.59 ± 6.01	0.291
NTCP_spinal cord_ (%)	2.45 × 10^−3^ ± 1.06 × 10^−2^	2.95 × 10^−3^ ± 1.35 × 10^−2^	0.532

PTV: planning target volume; TCP: tumor control probability; NTCP: normal tissue complication probability.

**Table 4:** Mean ± standard deviation of the planning evaluation indices in 10 test cases obtained from the dose distributions produced by the original and most usable beam arrangements.

	Original beam arrangement	Most usable beam arrangement	*P* value
PTV			
D95 (Gy)	45.5 ± 0.47	46.0 ± 0.60	0.029
Homogeneity index	1.13 ± 0.03	1.13 ± 0.04	0.643
Conformity index	1.70 ± 0.15	1.72 ± 0.17	0.376
TCP (%)	96.0 ± 0.27	96.1 ± 0.30	0.084
Lung			
V5 (%)	16.0 ± 6.30	14.7 ± 5.43	0.066
V10 (%)	9.96 ± 4.52	9.31 ± 3.53	0.161
V20 (%)	3.98 ± 1.46	4.03 ± 1.33	0.582
Mean dose (Gy)	3.03 ± 1.11	2.95 ± 1.03	0.152
NTCP_lung_ (%)	6.76 × 10^−3^ ± 1.22 × 10^−2^	5.40 × 10^−3^ ± 9.33 × 10^−3^	0.182
Spinal cord			
Maximum dose (Gy)	6.13 ± 3.62	7.09 ± 5.95	0.465
NTCP_spinal cord_ (%)	1.12 × 10^−5^ ± 1.92 × 10^−5^	4.37 × 10^−4^ ± 9.51 × 10^−4^	0.187
